# Management of chylous ascites following pancreaticoduodenectomy surgery using radiotherapy: A case report and review of literature

**DOI:** 10.1016/j.ijscr.2025.111640

**Published:** 2025-07-11

**Authors:** Mohammadsadra Shamohammadi, Alireza Ramezani, Ehsan Naseh, Alireza Gandomi-Mohammadabadi, Seyed Yahya Zarghami, Armaghan Abbasi Garavand

**Affiliations:** a Gastrointestinal and Liver Diseases Research Center, Iran University of Medical Sciences, Tehran, Iran; b General Surgery Department, School of Medicine, Iran University of Medical Sciences, Tehran, Iran; c Research Committee, Faculty of Medicine, Iran University of Medical Sciences, Tehran, Iran; d Division of HPB Surgery & Abdominal Organ Transplantation, Firoozgar Hospital, Iran University of Medical Sciences, Tehran, Iran; e School of Medicine, Iran University of Medical Sciences, Tehran, Iran

**Keywords:** Chylous ascites, Radiotherapy, Pancreatic cancer, Pancreaticoduodenectomy, Case report

## Abstract

**Introduction:**

Chylous ascites (CA) is a rare but significant clinical complication that requires careful consideration for effective treatment. CA often results from cisterna chyli injury following abdominal surgeries, especially pancreaticoduodenectomy (PD), due to triglyceride-rich lymphatic fluid accumulation in the peritoneal cavity. Management of CA ranges from conservative approaches to interventional strategies, particularly in refractory cases.

**Case presentation:**

A 76-year-old male who developed CA following PD for resectable ampulla of Vater cancer. Despite initial conservative treatments, including total parenteral nutrition (TPN), albumin supplementation, and octreotide administration, management of the patient's CA persisted with high-output ascitic drainage.

**Discussion:**

Although conservative management is often effective, it may fail in cases of high-output or persistent CA. Radiotherapy, by inducing localized fibrosis and sealing lymphatic leaks, represents a safe and efficacious option for refractory cases. The literature underscores the importance of multidisciplinary, stepwise management that incorporates conservative, interventional, and surgical modalities for optimal patient outcomes.

**Conclusion:**

CA is an uncommon and challenging postoperative complication of PD that requires a multidisciplinary management strategy. Although conservative management is the first-line approach, this case highlights the potential role of radiotherapy as an effective and safe adjunctive therapy for refractory cases.

## Highlights

• Chylous ascites is a rare and challenging complication following pancreaticoduodenectomy for pancreatic cancer.
• Initial conservative treatments were unsuccessful in managing the patient’s high-output ascitic drainage.
• Radiotherapy can be effectively used in the treatment of refractory chylous ascites.
• A multidisciplinary management strategy ensures optimal patient outcomes.

## 1 Introduction

CA is the pathological accumulation of lipid-rich lymphatic fluid in the peritoneal cavity [1]. It is an uncommon but important complication following extensive abdominal surgeries, especially those requiring lymphadenectomy, such as PD and other pancreatic resections [2]. The primary etiology is an intraoperative injury resulting in disruption of major lymphatic vessels including the cisterna chyli during lymph node dissection, which results in persistent lymphatic leakage into the peritoneal space [1,3].

The pathophysiology involves the disruption of normal lymphatic drainage, leading to leakage of chyle, a milky, triglyceride-rich lymphatic fluid produced after the absorption of dietary fats through the intestinal tract into the peritoneum [4]. Management strategies for postoperative chylous ascites are diverse, ranging from conservative measures to invasive procedures. Conservative therapy includes bowel rest with TPN [[4], [5], [6]], implementation of a medium-chain triglyceride (MCT) diet [7], albumin supplementation, and diuretics to reduce fluid volume [8,9]. Pharmacologic agents such as somatostatin analogs have also been shown to reduce lymphatic flow and promote the closure of lymphatic leaks [10]. If conservative management fails and the condition becomes refractory, interventional procedures are utilized to achieve resolution and facilitate leak closure [9,11,12].

The following review synthesizes findings from case reports, series, and key reviews, focusing on the pathophysiology, tumor associations, timing, and the comparative effectiveness of management strategies for chylous ascites after pancreatic and abdominal surgery. This case report has been written in accordance with the SCARE 2025 criteria [13].

## 2 Case presentation

A 76-year-old male was admitted to the emergency department with complaints of severe abdominal pain, weight loss, and jaundice. His medical history included hypertension, ischemic heart disease, and cerebrovascular accident. Clinical and imaging evaluation indicated resectable periampullary mass and pancreatic mass. Following the diagnostic workup, the patient was scheduled for a pylorus-preserving PD. Intraoperative findings revealed multiple lymphadenopathies, with notable lymph node involvement. The tumor exhibited invasion of both the superior mesenteric artery and the portal vein, necessitating complex dissection and extensive lymphadenectomy. The surgical pathology report confirmed moderately differentiated ductal adenocarcinoma in the head of the pancreas. The tumor measured 4 × 3.5 × 3 cm, with invasion into the duodenal wall and perineural involvement. Additionally, the pathology report noted the presence of five hilar and ten prepancreatic reactive lymph nodes. The patient had an uneventful initial postoperative course and was discharged in stable condition on postoperative day (POD) 7.

**Fig. 1:**
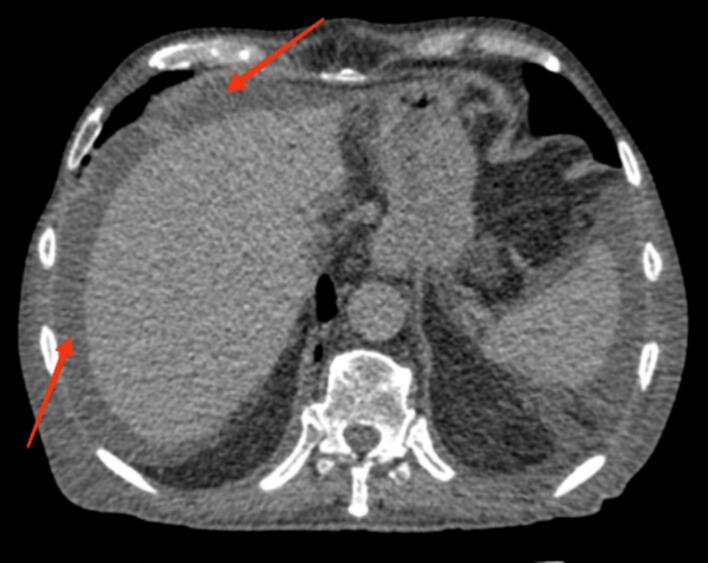
Axial CT scan demonstrating ascitic fluid accumulation in the upper abdomen, with evidence of the fluid surrounding the liver and other abdominal structures.

**Fig. 2:**
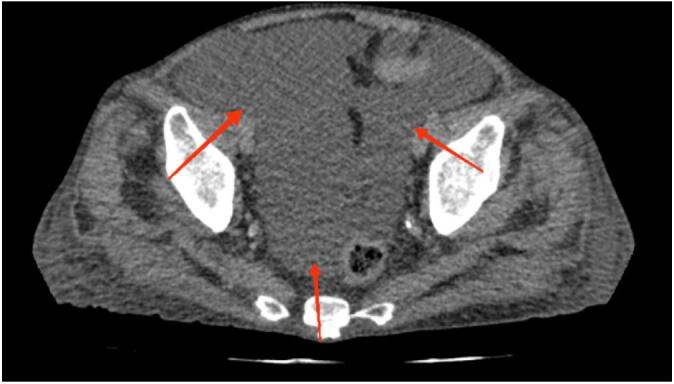
Axial CT scan showing the presence of ascitic fluid in the pelvic cavity, consistent with chylous ascites.

On POD 12, the patient developed abdominal pain, generalized weakness, and increased volume of fluid drainage. Over 2000 mL of slightly milky fluid was collected from the inserted abdominal drain, raising suspicion of CA. Biochemical analysis of the fluid confirmed the diagnosis showing elevated triglycerides and a lymphocyte-rich composition. The triglyceride level was measured at 350 mg/dL, which is consistent with the diagnostic criteria for CA. Also, computed tomography scan findings revealed the presence of ascites in the peritoneal and pelvic cavity (Fig. 1, Fig. 2).

Initial conservative management consisted of high-calorie TPN for 8 days, alongside ascites drainage and albumin replacement. Octreotide was administered to reduce portal pressure and improve lipid metabolism. Although ascite output decreased to 200 mL, the patient's symptoms worsened once the patient resumed an oral diet on 20 POD.

The patient underwent a second course of TPN for three weeks. During this second TPN course, a low-fat, MCT diet was also prescribed to minimize lymphatic stimulation. However, this did not lead to sufficient improvement and high-volume chylous drainage persisted.

On POD 41, due to the refractory nature of the chylous ascites, the patient underwent external beam radiotherapy. A total dose of 45 rGy was delivered in 25 daily fractions of 1.8 Gy using 6 megavoltage anterior-posterior conformal fields. The clinical target volume included the operative bed adjacent to the third and fourth portions of the duodenum, as well as regions at high risk for lymphatic leakage, including the cisterna chyli area, celiac axis, superior mesenteric artery, para-aortic lymphatic chains, and the portal vein zone. A 5 mm margin was added to form the planning target volume (PTV), and > 95 % of the PTV was covered by the 95 % isodose line. Organs at risk were contoured and dose constraints respected, including: small bowel D0.1cc < 45 Gy, bilateral kidney mean dose <15 Gy, spinal cord maximum <45 Gy, liver mean dose <28 Gy, and minimal exposure to uninvolved bowel loops, pancreas, and gastric antrum. Radiotherapy demonstrated remarkable efficacy, leading to a rapid and significant reduction in chylous ascites output. The patient's condition improved, allowing a gradual transition from TPN to an oral diet. During radiotherapy, the patient developed only mild nausea, which resolved with antiemetic therapy. No other gastrointestinal or hematologic toxicities were noted.

Two weeks after radiotherapy, the chylous drainage ceased completely, and the abdominal drain was successfully removed. The patient was gradually transitioned from TPN to an oral diet and eventually discharged on 55 POD in stable condition. During follow-up visits, no evidence of disease recurrence or ascites was detected on serial sonographic evaluations. Four months after discharge the patient unfortunately experienced a cardiac arrest and passed away.

## 3 Discussion

In the present study, we report a case of CA case following PD and investigate the management strategies for this condition. Our case involved an elderly male who developed refractory CA after PD, initially managed with conservative approaches including TPN, albumin supplementation, and octreotide. However, due to persistent high-output ascitic drainage, escalation to low-dose targeted radiotherapy was undertaken, leading to rapid and complete resolution of the ascites. This case, alongside a review of previously published reports (Table 1), allowed us to critically assess the available therapeutic options for postoperative CA, their mechanisms, clinical effectiveness, and overall impact on patient outcomes.

**Table 1:** Overview of cases of chylous ascites after pancreatic surgeries, detailing patient demographics, tumor types, timing, treatments, and cure durations.

First author (year)	Gender	Age (year)	Tumor type	Occurrence after surgery (day)	Treatment	Cure duration
M. Shamohammadi (2025)	M	74	Ampulla of Vater cancer	12	conservative management, TPN, radiotherapy	55
Al-Kubati (2023) [1]	F	65	PDAC	5	conservative management, TPN	21 days
B. S. Kim (2022) [2]		56	Pancreatic head cancer	19	conservative management, TPN, *Viscum album* (Mistletoe), Immunotherapy	114 days
T. K. Nguyen (2021) [3]	M	59	PDAC	7	lymphangiography, Sclerotherapy, TPN	5 weeks
Nguyen, T. K. (2021) [4]	F	59	PDAC	20	lymphangiography, Sclerotherapy, TPN	17 days
T. K. Nguyen (2021) [4]	M	59	PDAC	NA	conservative management, lymphangiography, Sclerotherapy	NA
T. K. Nguyen (2021) [4]	M	73	PDAC	5	conservative management, lymphangiography	21 days
T. K. Nguyen (2021) [4]	F	71	PDAC	5	conservative management, lymphangiography	21 > days
Hasegawa, T. (2021) [5]	M	62	intraductal papillary mucinous neoplasm	7	conservative management, lymphangiography, Embolization	80 days
Takahashi, Y. (2020) [6]	F	84	cholangiocarcinoma	4	conservative management	24 days
Yokota, T. (2019) [7]	F	67	PDAC	NA	conservative management	NA
Smith, E. B. (2019) [8]	F	1.5	inflammatory myofibroblastic tumor	NA	TPN	NA
Hirata, M. (2017) [9]	F	71	extrahepatic bile duct cancer	20	lymphangiography, conservative management	13 days
Ignjatović, M. (2016) [10]	F	55	extramedullary plasmacytomas of the duodenum	0	conservative management	5 days
Poves, I. (2015) [11]	F	77	PDAC	0	conservative management	17 days
Corradini, S. (2015) [12]	M	76	Pancreatic head cancer	0	conservative management, TPN, radiotherapy	6 weeks
Kim, C. (2011) [13]	F	70	Pancreatic head cancer	NA	NA	NA
D'Hondt, M. (2011) [14]	M	62	periampullary polyp and adenocarcinoma	NA	Denver shunt, lymphangiography	5
Plummer, J. M. (2010) [15]	F	21	Duodenal fibrosarcoma	0	conservative management, TPN	4 weeks

Abbreviations: PDAC: Pancreatic ductal adenocarcinoma/TPN: Total parenteral nutrition/HLG: Hepatic lymphangiography/NA: Not applicable or not available.

CA is a rare but serious complication after pancreatic surgery, with an incidence of up to 11 % depending on the extent of lymphadenectomy [4]. CA results from the disruption of abdominal lymphatic structures, mainly the cisterna chyli, during surgical dissection [1]. The cisterna chyli collects lymph from the abdominal and lower body and drains into the thoracic duct; injury leads to leakage of triglyceride-rich, milky chyle into the peritoneal cavity [14]. This fluid contains high levels of triglycerides (>200 mg/dL) and lymphocytes, providing diagnostic clues [15]. CA significantly impacts postoperative recovery, nutritional status, and survival, requiring careful management strategies [16].

First-line management commonly begins with TPN or MCT to minimize lymphatic stimulation, promoting spontaneous closure of the leak [17]. TPN effectively reduces chyle flow by completely bypassing the gut, but prolonged use (>2 weeks) risks infections and hepatic dysfunction [18]. Supplementation with MCT diets, which are absorbed directly into the portal vein rather than intestinal lymphatics, further assists recovery. Yoshida et al. [7] demonstrated that an MCT-based diet improved the success rate of conservative management in CA patients significantly.

Somatostatin analogues (e.g., octreotide) are used as an adjuvant treatments, acting via inhibition of lymphatic flow and gastrointestinal secretions [19]. Takahashi et al. [10] showed that octreotide administration reduced chyle production and facilitated leak closure without adverse effects. Mechanistically, somatostatin reduces splanchnic blood flow and lymphatic pressure, thereby promoting leak healing. Additionally, Takahashi et al. [20] reported complete remission of CA with etilefrine, hypothesizing that it increases venous and lymphatic smooth muscle tone, thus reducing lymphatic leak.

Lymphangiography serves both diagnostic and therapeutic roles, enabling targeted embolization with agents like n-butyl-2-cyanoacrylate (NBCA) [21]. Kim et al. [22] and Corradini et al. [23] reported rapid resolution of CA following NBCA embolization after lymphangiographic localization. Lymphangiography also offers the opportunity for sclerotherapy using agents like the *Viscum album*, as described by Lee et al. [24], particularly effective for small lymphatic fistulas. Sclerotherapy promotes the closure of lymphatic leaks by inducing local inflammatory fibrosis and has proven beneficial, especially in cases where embolization alone is insufficient [25]. Also, D'Hondt et al. [11] reported a case in which Denver shunt placement was utilized as an alternative for continuous drainage of prolonged CA.

Radiotherapy induces localized fibrosis in leaking lymphatic channels via low-dose external beam radiation, effectively sealing the leak [26]. Previous reports have demonstrated resolution of leaks with doses as low as 8–10 Gy with 1 Gy fractions administrated daily [[27], [28], [29], [30]]. Brown et al. [28] prescribed 10 Gy in daily 1 Gy fractions targeting the cisterna chyli/thoracic duct (T12–L2) in a patient with refractory chylous ascites. The leak closed with no recurrence and no acute toxicity. In our case, because of persistent high-output drainage, we used a more definitive regimen of 45 Gy in 25 fractions, with target volumes encompassing the operative bed and high-risk lymphatic pathways. The patient tolerated therapy well, with rapid resolution of ascites and no significant toxicity. These results support radiotherapy as a viable adjunct when conservative and interventional strategies fail. Furthermore, Corradini et al. [23] reported the successful closure of persistent lymphatic leaks following PD using radiation. Although there are concerns about potential adverse effects (e.g. intestinal fibrosis), radiotherapy has been proven to be safe and effective [31]. Radiotherapy dose–volume guidelines confirm that cardiac toxicity is associated with direct exposure and typically manifests years after treatment [32,33]. In our case, no acute or late radiation toxicity was observed. The patient did have pre-existing ischemic heart disease. The patient's cardiac arrest four months post-radiotherapy is unlikely to be treatment-related. The heart was anatomically excluded from the radiation field, which was confined to the upper abdomen.

## 4 Conclusion

This case demonstrates that CA following PD is a rare but serious postoperative complication requiring a comprehensive, multidisciplinary management approach. Although conservative management is the initial treatment, refractory cases need supplementary therapies to achieve a better outcome. Although first-line treatments like TPN is typically effective, radiotherapy demonstrated remarkable efficacy and safety in the management of ascites in our case.

## Abbreviations

CA: Chylous Ascites
TPN: Total Parenteral Nutrition
MCT: Medium-Chain Triglycerides
PD: Pancreaticoduodenectomy
POD: Postoperative Day
NBCA: n-butyl-2-cyanoacrylate
NA: Not applicable
PTV: planning target volume

## Consent

Informed written consent was obtained from the patient for publication of this case report and any accompanying images. A copy of the written consent is available for review by the Editor-in-Chief of this journal upon request.

## Ethical approval

Ethical approval by the Research Committee was not necessary, as the format of this article is a case report.

## Funding statement

Not applicable.

## Declaration of competing interest

All authors declare that there are no conflicts of interest related to this study.
